# Nanozymes with Multiple Activities: Prospects in Analytical Sensing

**DOI:** 10.3390/bios12040251

**Published:** 2022-04-16

**Authors:** Xiangheng Niu, Bangxiang Liu, Panwang Hu, Hengjia Zhu, Mengzhu Wang

**Affiliations:** 1 State Key Laboratory of Urban Water Resource and Environment, Harbin Institute of Technology, Harbin 150090, China; 2 Institute of Green Chemistry and Chemical Technology, School of Chemistry and Chemical Engineering, Jiangsu University, Zhenjiang 212013, China; lbx15912338469@163.com (B.L.); wc2337556161@163.com (P.H.); 18260622650@163.com (H.Z.); wangmengzhu2018@126.com (M.W.); 3 Jiangsu Provincial Key Laboratory of Environmental Science and Engineering, Suzhou University of Science and Technology, Suzhou 215009, China

**Keywords:** nanozyme, multiple activities, cascade catalysis, analytical application, sensing

## Abstract

Given the superiorities in catalytic stability, production cost and performance tunability over natural bio-enzymes, artificial nanomaterials featuring enzyme-like characteristics (nanozymes) have drawn extensive attention from the academic community in the past decade. With these merits, they are intensively tested for sensing, biomedicine and environmental engineering. Especially in the analytical sensing field, enzyme mimics have found wide use for biochemical detection, environmental monitoring and food analysis. More fascinatingly, rational design enables one fabrication of enzyme-like materials with versatile activities, which show great promise for further advancement of the nanozyme-involved biochemical sensing field. To understand the progress in such an exciting field, here we offer a review of nanozymes with multiple catalytic activities and their analytical application prospects. The main types of enzyme-mimetic activities are first introduced, followed by a summary of current strategies that can be employed to design multi-activity nanozymes. In particular, typical materials with at least two enzyme-like activities are reviewed. Finally, opportunities for multi-activity nanozymes applied in the sensing field are discussed, and potential challenges are also presented, to better guide the development of analytical methods and sensors using nanozymes with different catalytic features.

## 1. Introduction

As a class of biocatalysts with high activity and excellent specificity toward substrates, natural enzymes have been extensively employed in various areas including biomedicine, food processing and agricultural engineering, as well as environmental protection. Nonetheless, the protein nature of most natural enzymes makes it very easy to decrease or even completely lose their catalytic ability in harsh environments. In addition, the complex extraction and purification processes for these bio-enzymes greatly increase their production and use costs. In this context, scientists have been trying to develop artificial materials with enzyme-like catalytic characteristics but possessing good stability and low cost [1,2,3].

Since Yan’s pioneering group reported that common Fe_3_O_4_ magnetic nanoparticles (Fe_3_O_4_ MNPs) could exhibit catalytic characteristics similar to natural horseradish peroxidase (HRP) [4], abundant attention and interest have been given to nanomaterials with enzyme-mimetic features (defined as “nanozymes”) [5,6,7,8]. In comparison with natural bio-enzymes, these artificial nanozymes can present excellent stability under harsh conditions and with a lower production cost. More importantly, their catalytic performance can be rationally designed and tailored via the engineering of composition, size, shape, crystal, etc. As a consequence, nanozymes are recognized as promising alternatives to the corresponding bio-enzymes and have found intensive applications in the fields of sensing [9,10], biomedicine [11,12], environmental remediation [13,14,15] and food science [16,17]. Especially in the biochemical sensing field, signal amplification enabled by nanozyme catalysis makes it possible to detect analytes with high sensitivity [18,19]. These can be used as bio-enzyme alternatives to fabricate cascade sensing systems [20] and to develop nanozyme-linked immuno-sorbent assays [21]. The surface regulation of nanozymes by DNA chains also enables the design of catalytic apta-sensors [22]. Besides, potential interactions between analytes and nanozymes make it possible to explore various detection methods and sensors for ions and small molecules [23,24,25,26,27,28,29].

In the last decade, although various artificial materials featuring peroxidase-, oxidase-, catalase- or superoxide dismutase-like catalytic characteristics have been explored and applied, in real biological systems multi-enzyme complexes are usually involved in order to catalyze cascade reactions. To better mimic these systems, scientists are engaged in designing nanozymes that can exhibit multiple activities simultaneously under the same or similar conditions [30,31,32,33,34]. For analytical sensing, the use of multi-activity nanozymes makes it easier to fabricate cascade sensing systems. What is more, it not only expands the scope of analytes but also improves detection performance, because cascade catalysis occurring on a multi-enzyme-like catalyst can offer beneficial spatial proximity and shorter mass diffusion for efficient sensing compared to that on two or more single-enzyme-like catalysts. Additionally, in some cases multi-activity nanozymes can simplify the operation for detecting targets. Within this context, nanozymes with multiple activities are being designed to further advance analytical applications [35,36,37,38,39]. However, the use of nanozymes with multiple catalytic features may also bring some potential problems for biochemical sensing, which should not be neglected. For instance, both peroxidase- and oxidase-like activities can catalyze the oxidation of chromogenic substrates to give color signals for detection, and the unavoidable oxidase-like catalytic reaction will affect the detection of an analyte based on the peroxidase-mimetic activity. Therefore, both opportunities and challenges exist for multi-activity nanozymes used in the analytical sensing field.

**Figure 1:**
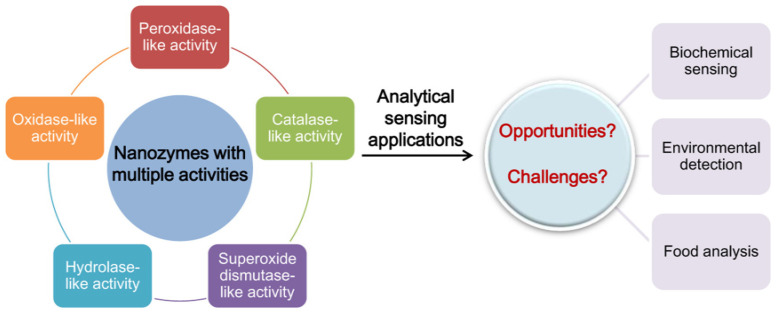
Schematic diagram of nanozymes with multiple catalytic activities and their applications in the analytical sensing field.

To highlight progress in this exciting field, here we offer a review of multi-activity nanozymes and their analytical application prospects (Figure 1). What should be noted is that, although many excellent reviews of nanozymes and their versatile applications have been reported [5,6,7,8,11,40,41,42], none thematically introduces nanozymes featuring multiple activities, and their opportunities and challenges in analytical sensing. In this review, typical types of enzyme-mimicking activities are first introduced in detail, followed by summarizing strategies available for designing multi-activity nanozymes. Next, current materials exhibiting at least two enzyme-mimetic activities are emphatically reviewed. In the end, applications and potentials of multi-activity nanozymes in the bio-sensing field are discussed, and corresponding challenges are also presented. This contribution is expected to deepen the understanding of nanozymes with more than one catalytic activity, and will also provide a guide for better development of analytical methods and sensors using multi-activity nanozymes.

## 2. Types of Enzyme-like Catalytic Activities

To date, most of the effort from the nanozyme community has been put into developing materials with oxidoreductase-mimetic features, including peroxidase, catalase, oxidase, and superoxide dismutase. Apart from these oxidoreductases, scientists are engaged constantly in exploring new enzyme-like types, and some hydrolase mimics have also been reported.

### 2.1. Peroxidase Mimics

**Figure 2:**
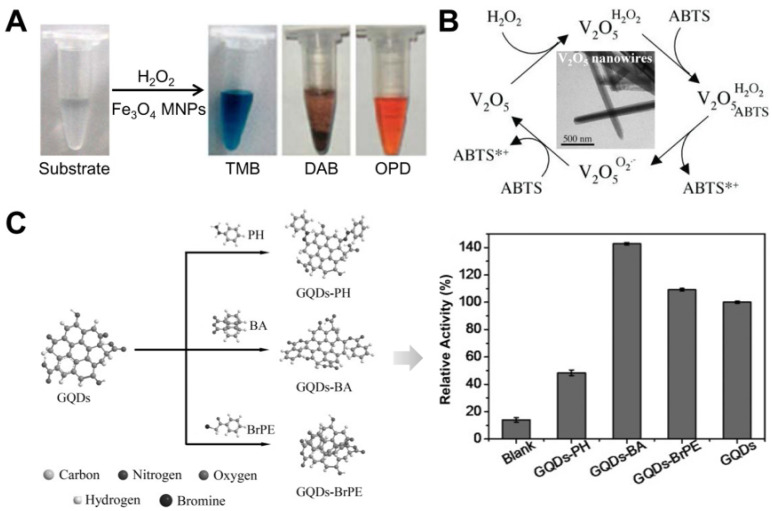
(**A**) demonstrates the peroxidase-mimicking activity of Fe_3_O_4_ MNPs catalyzing the chromogenic reactions of different substrates with the participation of H_2_O_2_ (Reprinted with permission from Ref. [4]. Copyright 2007, Springer Nature). (**B**) presents a possible mechanism for peroxidase-mimetic V_2_O_5_ nanowires triggering the redox reaction between H_2_O_2_ and ABTS (Reprinted with permission from Ref. [43]. Copyright 2011, John Wiley & Sons). (**C**) illustrates the regulation of GODs featuring different chemical groups on their surface and peroxidase-mimetic activities (Reprinted with permission from Ref. [44]. Copyright 2015, John Wiley & Sons).

As noted in Equation (1), peroxidase is a kind of natural enzyme with the capacity to transform H_2_O_2_ into H_2_O with the participation of a reductive substrate (AH_2_), where the reductive substrate AH_2_ is catalytically oxidized to its corresponding oxide (A). As the most common peroxidase, HRP is widely applied in H_2_O_2_-related catalysis, sensing and biomedicine. In 2007, Yan’s group found that common Fe_3_O_4_ MNPs exhibited a catalytic behavior in order to trigger the chromogenic reactions between H_2_O_2_ and different substrates [4]. In detail, the colorless substrate 3,3′,5,5′-tetramethylbenzidine (TMB) could be catalytically converted into a blue product in the presence of H_2_O_2_ (Figure 2A). When di-azo-amino-benzene (DAB) or *o*-phenyl-enediamine (OPD) was used as the substrate instead, corresponding color changes were also observed. The result revealed that the Fe_3_O_4_ particles, similarly to HRP, showed catalytic activity toward these typical substrates. Further study found that the catalytic reaction followed a typical Michaelis-Menten kinetic process, and a ping-pong mechanism could be used to explain the peroxidase-like reaction. Use of Fe_3_O_4_ particles as an alternative to HRP in immunoassays was also validated in [4].

(1) $${\mathrm{AH}}_{2}+{\;H}_{2}O_{2}\;\to \;A\;+2H_{2}O$$

Since then, a large number of Fe-based peroxidase mimics have been intensively explored, and the material category has also been expanded to other transition metals [43,45,46,47,48,49,50] and noble metals [51,52,53,54]. Typically, André et al. developed V_2_O_5_ nanowires as an intrinsic vanadium-dependent halo-peroxidase (V-HPO) mimic [43], and speculated about a possible catalytic mechanism for the material according to its layered orthorhombic structure. In the material, the V atoms in the (010) face provided Lewis acid sites, whereas the electron lone pairs of the bridging O atoms acted as Lewis base sites. As a result, the V_2_O_5_ nanowires initially combined with H_2_O_2_ by forming an intermediate peroxo species (Figure 2B). In the subsequent step, the substrate 2,2-azino-bis(3-ethylbenzothiazoline-6-sulfonic acid) (ABTS) could bind to the intermediate peroxo species by a nucleophilic attack, allowing the oxidation of colorless ABTS to its green product ABTS*^+^. Given that H_2_O_2_ was a two-electron oxidant, another ABTS molecule was needed to allow the regeneration of the V-HPO mimic.

Interestingly, some carbon-based materials without redox metal species have also been found to exhibit peroxidase-mimicking catalytic activity [44,55]. Song et al. observed that carboxyl-modified graphene oxide (GO-COOH) could show an intrinsic peroxidase-mimetic ability to catalyze the oxidation of TMB with the participation of H_2_O_2_ in order to produce a blue species [55], and they further fabricated a cascade catalytic system by integrating the artificial peroxidase mimic GO-COOH with natural glucose oxidase (GOx) for the colorimetric detection of glucose. To better understand such a peroxidase-like activity, the same group took graphene quantum dots (GODs) as a model and applied the chemical titration method to manipulate the surface groups on GODs [44]. In detail, three different reagents, namely phenyl-hydrazine (PH), benzoic anhydride (BA) and 2-bromo-1-phenylethanone (BrPE), were used to consume as much as possible the ketonic carbonyl, hydroxyl and carboxylic groups, respectively (Figure 2C). As a result, it was found that the GQDs-BA formed showed the highest catalytic activity, the activity of the formed GQDs-PH was much lower than that of original GQDs, and the activity of the formed GQDs-BrPE showed no obvious change compared to GODs. It was concluded that the ketonic carbonyl groups acted as the catalytically active sites, the carboxylic groups offered the substrate-binding sites, and the hydroxyl groups could inversely inhibit such an intrinsic peroxidase-mimicking activity. This conclusion provides us with some guides to design and develop carbon-based peroxidase mimics with both high activity and good substrate affinity.

### 2.2. Catalase Mimics

Catalase is a type of enzyme having the ability to catalyze the decomposition of H_2_O_2_ into O_2_ and H_2_O (Equation (2)). With such a capacity, catalase is often employed to remove excess reactive oxygen species H_2_O_2_ or to provide on-demand O_2_ for cancer therapy and cyto-protection. Currently, catalase-mimicking catalytic activity has been found in some Fe [56,57], Cu [58], Co [59] and noble metal [60,61,62] materials.

(2) $$2H_{2}O_{2}\;\to {\;O}_{2}+2H_{2}O\;$$

Different from peroxidase that exhibits the best catalytic activity under acidic conditions, catalase usually presents this activity in basic solutions. To explain such a difference, Gao’s group performed a detailed computational study [62]. Here, taking Au as a typical example, in acidic solutions with H pre-adsorbed on the Au(111) surface, H_2_O_2_ could further be adsorbed onto the surface, and a base-like decomposition pathway of the adsorbed H_2_O_2_ occurred to form adsorbed OH* and H_2_O*, followed by the conversion of adsorbed OH* into O* and H_2_O*. When the active species O* further attacked the substrates, a peroxidase-mimetic catalytic process was completed. Instead, under basic conditions with OH pre-adsorbed on the Au(111) surface, the substrate molecule H_2_O_2_ would firstly transfer an H atom to the pre-adsorbed OH to produce H_2_O* and HO_2_*. After that, the absorbed HO_2_* gave an H atom to another H_2_O_2_, finally generating O_2_* and H_2_O*. As a consequence, the catalase-mimicking activity of the noble metal was preferably observed in alkaline solutions.

### 2.3. Oxidase Mimics

Apart from peroxidase and catalase, some materials have been explored as oxidase mimics, including Mn- [63,64,65], Cu- [66,67], Ni- [68], Co- [69], Ce- [70] and Fe-based [71] compounds. These can directly catalyze the oxidation reaction of substrates in the absence of H_2_O_2_. Instead, O_2_ dissolved in solutions is usually consumed in the oxidase-like reaction (Equation (3)). In comparison with peroxidase, oxidase mimics are preferable in some cases for biochemical analysis, because no addition of the substrate H_2_O_2_ is required, and its potential impact on sensing can also be avoided [9].

(3) $${\mathrm{AH}}_{2}+{\;O}_{2}\;\to \;A\;+{\;H}_{2}O_{2}$$

**Figure 3:**
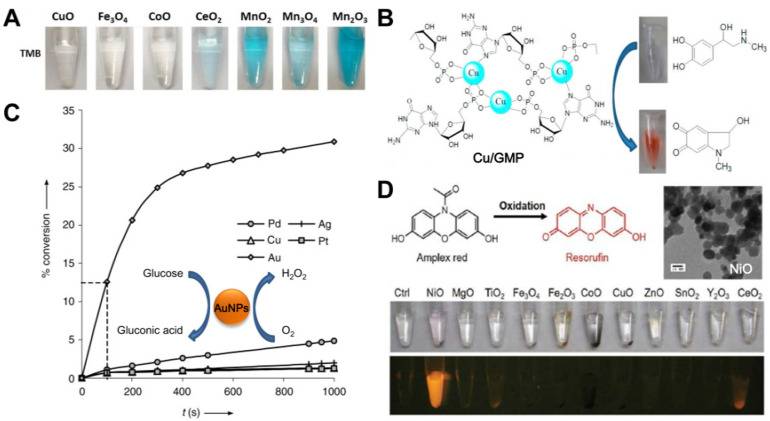
(**A**) compares the dissolved oxygen-mediated TMB oxidation reaction catalyzed by various metal oxides (Reprinted with permission from Ref. [65]. Copyright 2021, John Wiley & Sons). (**B**) illustrates the laccase-like feature of Cu/GMP catalyzing the oxidation of epinephrine (Reprinted with permission from Ref. [67]. Copyright 2017, American Chemical Society). (**C**) compares the catalytic activity of various metal particles toward glucose oxidation (Reprinted with permission from Ref. [73]. 2004, John Wiley & Sons). (**D**) demonstrates that NiO particles can catalyze the oxidation of Amplex red to fluorescent resorufin specifically (Reprinted with permission from Ref. [68]. Copyright 2018, Royal Society of Chemistry).

Typically, Mn-based materials can show good oxidase-like activity due to the presence of multi-valence Mn species (Mn^2+^, Mn^3+^ and Mn^4+^) [63,64,72]. In a detailed study performed by Chen et al. [65], different metal oxides, including CuO, Fe_3_O_4_, CoO, CeO_2_, MnO_2_, Mn_3_O_4_ and Mn_2_O_3_, were tested to catalyze the oxidation of TMB without the addition of H_2_O_2_. It was found that all the Mn oxides could trigger the color reaction of TMB (Figure 3A), but they exhibited different catalytic activities. Among the three Mn oxides, Mn_2_O_3_ performed the strongest oxidase-like activity under identical conditions.

As a special oxidase with the ability to catalyze the oxidation of phenolic compounds to corresponding products, Cu-containing laccase is usually utilized to degrade organic pollutants. Inspired by natural Cu-containing laccase, Liu’s group explored guanosine monophosphate (GMP) coordinated Cu^2+^ as an artificial laccase mimic [67]. It was found that the Cu/GMP formed with a Cu-to-GMP ratio of 3:4 could present a higher catalytic activity than natural laccase to trigger the conversion of epinephrine to benzoquinone (Figure 3B), and stable performance under various harsh conditions was also observed in the laccase mimic.

In addition to the above transition metal compounds, some noble metals have also been found to present unique oxidase-like features [73,74,75]. Comotti et al. observed that “naked” gold nanoparticles (AuNPs) could catalytically oxidize glucose with the participation of dissolved O_2_ [73], very similar to natural GOx when catalyzing glucose into gluconic acid by utilizing O_2_ as an electron acceptor with the simultaneous formation of H_2_O_2_. In contrast, other metal particles tested, including Pd, Ag, Cu and Pt, did not show significant oxidase-like activity under the same condition (Figure 3C). A recent detailed study further demonstrated that the reaction path of glucose oxidation catalyzed by AuNPs was the same as that of natural GOx [75], except that OH^-^ was preferably used as a Brønsted base to abstract H^+^ from the substrate glucose. Inspired by this initial finding, Luo et al. reported a self-catalyzed self-limiting system for the controllable growth of GOx-like AuNPs [74]. In the system, the AuNPs served as both a GOx-like catalyst and seeds for Au growth. In detail, when glucose was fed, the AuNPs first acted as a GOx mimic to catalyze the oxidation of glucose, producing the intermediate H_2_O_2_ in situ, which then could induce the seeded growth of AuNPs with the addition of auric chloride ions. Interestingly, the growth of AuNPs was internally regulated by two negative feedback factors, namely the size-dependent decrease in activity of AuNPs and the gluconic acid-triggered surface passivation, thus resulting in the self-limiting catalytic and growth system of AuNPs.

Particularly, Li et al. tried to use various commercial metal oxide particles to trigger chromogenic or fluoro-genic reactions with no addition of H_2_O_2_ [68], and they observed that NiO particles could, remarkably, induce the conversion of Amplex red to fluorescent resorufin (Figure 3D), while other metal oxides had no similar capacity, indicating the oxidase-like catalytic behavior of NiO particles toward the substrate. Interestingly, the material only made response to the Amplex red substrate but not to other substrates, such as TMB and ABTS. Excellent catalytic activity at physiological pH and low cytotoxicity were also observed in the NiO particles, implying their promising applications in fluorescence sensing and imaging.

### 2.4. Superoxide Dismutase Mimics

Superoxide dismutase is a type of enzyme capable of catalyzing the disproportionate reaction of unstable superoxide radicals to H_2_O_2_ and O_2_ (Equation (4)). Similar to catalase, superoxide dismutase is often used to eliminate excess reactive oxygen species for cyto-protection and cancer therapy. Currently, the superoxide dismutase-like activity has been observed in Ce-based [76,77] and Cu-based [58,78,79] materials.

(4) $$2O_{2}^{-}+2H^{+}\;\to {\;O}_{2}+{\;H}_{2}O_{2}$$

Typically, Zhang et al. developed a Cu-based framework material that exhibited notable superoxide dismutase-mimicking activity [79]. The material was composed of Cu^2+^ and tetra-carboxylic phenyl porphyrin (TCPP), with active Cu sites coordinated by N and O atoms, very similar to the coordination environment of natural superoxide dismutase. To further enhance the superoxide dismutase-mimetic catalytic activity of the Cu-TCPP sheets, they were ultrasonically tailored into smaller nano-dots. In comparison with sheet-structured Cu-TCPP, these dots had a similar size to natural enzymes and could provide more accessible catalytic sites to substrates with lower diffusion barriers. As validated by in vitro and in vivo experiments, the fabricated Cu-TCPP nano-dots exhibited an impressive catalytic efficiency as an artificial superoxide dismutase mimic in alleviating endo-toxemia.

### 2.5. Hydrolase Mimics

**Figure 4:**
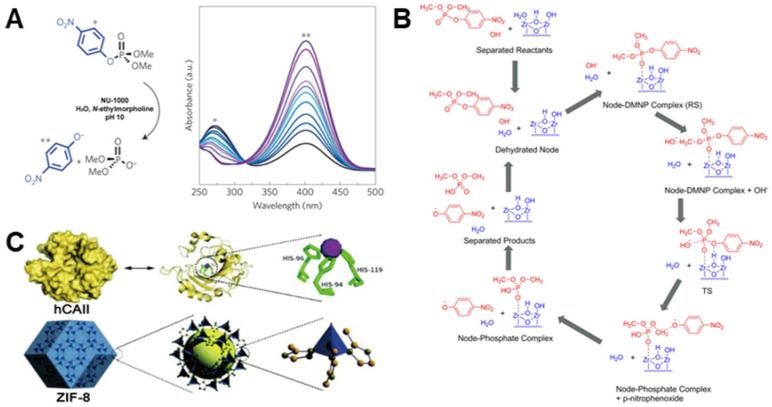
(**A**) reveals the hydrolase-like catalytic feature of Zr-containing NU-100 decomposing DMNP (Reprinted with permission from Ref. [80]. Copyright 2015, Springer Nature). (**B**) illustrates the catalytic cycle of DMNP hydrolysis on an NU-1000 node (Reprinted with permission from Ref. [84]. Copyright 2018, American Chemical Society). (**C**) displays that Zn-containing ZIF-8 has a similar structure to natural hCAII and can show hydrolytic activity toward *p*NPA (Reprinted with permission from Ref. [85]. Copyright 2019, Royal Society of Chemistry).

Hydrolases are a class of natural enzymes that can trigger various hydrolysis reactions. So far, there are three main categories of hydrolases that artificial inorganic materials can mimic according to hydrolytic substrate [80,81,82,83,84,85,86,87,88,89]: organophosphorus hydrolase, esterase, and protease. For instance, several Zr-based materials have demonstrated the capacity of breaking the phosphonate ester bond [80,81,82,83,84]. Typically, Mondloch et al. designed a Zr-based metal-organic framework (MOF), NU-1000, to hydrolyze chemical warfare agents containing the phosphonate ester bond [80]. The NU-1000 was composed of eight connected Zr_6_(μ_3_-O)_4_(μ_3_-OH)_4_(H_2_O)_4_(OH)_4_ nodes and tetra-topic 1,3,6,8(*p*-benzoate)pyrene linkers, and offered rich pores and channels to allow phosphate ester molecules to permeate into the whole framework for catalysis. When using dimethyl 4-nitrophenyl phosphate (DMNP) as a typical substrate, it was hydrolyzed to phosphate and *p*-nitro-phenoxide anion in medium alkaline solutions under the catalysis of the proposed NU-1000 (Figure 4A). Further study revealed that the hydrolase-mimetic activity of the Zr-based material was due to the specific interaction between Zr-O clusters it contained and the phosphate group in enzymatic substrates (Figure 4B) [84].

Recently, inspired by the structure and composition of human carbonic anhydrase II (hCAⅡ), Chen et al. developed several zeolitic imidazolate frameworks (ZIFs) that could catalyze the hydrolysis of esters [85]. In natural hCAII, its active center was composed of a tetra-hedrally coordinated Zn^2+^ with three symmetric histidine imidazoles and a H_2_O (Figure 4C). Similar to natural hCAII, the designed ZIF-8 was composed of a central Zn^2+^ and four 2-methylimidazolate (2-mIM) ligands, where the tetra-hedrally coordinated Zn^2+^ was interconnected through 2-mIM to form a porous framework structure. As a result, the developed material presented an esterase-like catalytic ability comparable to natural hCAII in order to trigger the hydrolysis of *p*-nitro-phenyl acetate (*p*NPA).

Apart from organophosphorus hydrolase and esterase, artificial materials containing Cu active sites have been found to present hydrolysis capacity toward proteins [88,89]. Li et al. explored two kinds of Cu-containing MOFs as potential protease mimics [88], and it was found that the Cu_2_(C_9_H_3_O_6_)_4/3_ MOF, also known as HKUST-1, could offer a stronger affinity toward the protein substrate bovine serum albumin (BSA), with a Michaelis-Menten constant (*K*_m_) 26,000-fold smaller than that of natural trypsin.

## 3. Nanozymes with Multiple Activities

In addition to the above-mentioned mimics featuring single-enzyme catalytic activity, more and more inorganic materials are found to have the ability to show multiple activities in different environments, or even under the same condition. This attractive feature, with two or more enzyme-mimetic activities in one catalyst, enables an easier design of cascade catalytic systems for biomedicine and sensing [34,39,90]. As a typical example, a Cu-based MOF consisting of Cu^2+^ active nodes and tereph-thalic acid (TA) linkers was designed by our group and provided both cysteine oxidase- and peroxidase-mimicking activities [39]. In the MOF, the Cu-O clusters not only catalyzed the redox reaction of cysteine and dissolved O_2_ with the simultaneous formation of H_2_O_2_, but also provided peroxidase-mimetic activity to convert the intermediate H_2_O_2_ into hydroxyl radicals under the same condition, which further oxidized the TA ligands to a fluorescent species. With the cascade reaction catalyzed by the Cu-MOF, together with its stimulus-responsive fluorescence, an easy method was established with simple operation and high performance for cysteine determination. This example implies great promise for multi-enzyme-mimetic materials in related applications. In the next part, the strategies available for the design of nanozymes with multiple activities will be summarized, and typical multi-enzyme-mimetic materials will also be highlighted.

### 3.1. Strategies to Design Nanozymes with Multiple Activities

**Figure 5:**
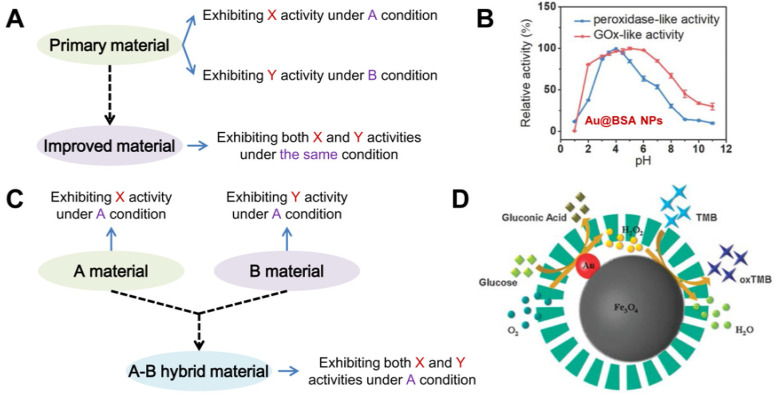
(**A**) shows the strategy for improving primary materials featuring different enzyme-like activities under different conditions to obtain nanozymes with multiple activities under the same condition. (**B**) demonstrates both the peroxidase- and GOx-like activities of Au@BSA NPs under similar pH conditions (Reprinted with permission from Ref. [36]. Copyright 2018, John Wiley & Sons). (**C**) illustrates the strategy of hybridizing materials together to obtain nanozymes with multiple activities under the same condition. (**D**) presents the cascade catalytic system based on the GOx- and peroxidase-like activities of Fe_3_O_4_-Au@MS (Reprinted with permission from Ref. [38]. Copyright 2013, Royal Society of Chemistry).

Currently, there are two main strategies that can be employed to design nanozymes with multiple activities. The first is to improve the primary materials that exhibit different activities under different conditions (Figure 5A). As found in the above examples, many materials can provide peroxidase-like activity and catalase-, oxidase- or superoxide dismutase-mimicking catalytic behavior, but these activities are often observed in different environments. For example, peroxidase-mimetic catalytic processes are usually completed in medium acidic solutions, whereas only in alkaline environments can the catalase-like activity be presented. In this regard, a variety of means, including surface modification, support engineering, and size adjustment, have been developed to regulate the catalytic behaviors of nanozymes in various pH environments [91,92,93]. These means, theoretically, can be used to improve primary materials, in order to obtain nanozymes featuring multiple activities under similar or the same condition. Typically, “naked” Au NPs can show peroxidase-like activity and GOx-like catalytic behavior, but the optimal pH ranges of the both activities are obviously different. To this end, Zhang et al. designed “non-naked” Au NPs that could present the dual enzyme-like activities at the same pH [36]. They used BSA as a stabilizer and protector to prepare the Au NPs. It was observed that the Au@BSA NPs could exhibit notable peroxidase-mimetic activity in a wide pH scope and retained over 90% catalytic activity in the pH range 3.5–4.5 (Figure 5B). For the GOx-like activity, the Au@BSA NPs retained over 90% activity in the pH scope from 3.0 to 6.0. According to the result, the peroxidase- and GOx-like activities of the Au@BSA NPs had an overlapping pH range. By selecting a pH 4.0 solution as the reaction medium, a tandem enzyme-like catalytic system was successfully established, where the Au@BSA NPs could first catalyze the oxidation of glucose to produce H_2_O_2_ and then catalyze the intermediate H_2_O_2_ to H_2_O in the same environment.

The second way to acquire multi-enzyme-mimetic nanozymes is to hybridize two or more materials together, featuring different activities under similar conditions. As illustrated in Figure 5C, there are a number of single-component materials that can exhibit various catalytic behaviors in a similar or the same environment. By rationally combining these single-component entities together, the formed hybrid material may exhibit two or more enzyme-like activities under the same condition. With such a strategy, several multi-activity nanozymes have been rationally designed for cascade catalysis [14,38,94,95]. Typically, peroxide-like Fe_3_O_4_ and GOx-like AuNPs were integrated to form a composite that exhibited both peroxidase- and GOx-mimetic activities in the same system [38]. As illustrated in Figure 5D, the small Au NPs part in the mesoporous silica-coated Fe_3_O_4_-Au hybrid (Fe_3_O_4_-Au@MS) possessed intrinsic GOx-mimetic activity and could catalyze the oxidation of glucose with dissolved O_2_, producing gluconic acid and H_2_O_2_. The produced H_2_O_2_ was confined in the porous microsphere, and then was directly catalyzed by the Fe_3_O_4_ part of the hybrid as a peroxidase mimic, thus inducing the remarkable chromogenic reaction of TMB.

### 3.2. Typical Nanozymes with Multiple Activities

Thanks to the above strategies, a number of artificial enzymes with multiple activities have been fabricated [39,90,96,97,98,99,100,101,102,103]. Here, several typical multi-activity nanozymes are introduced in detail.

**Figure 6:**
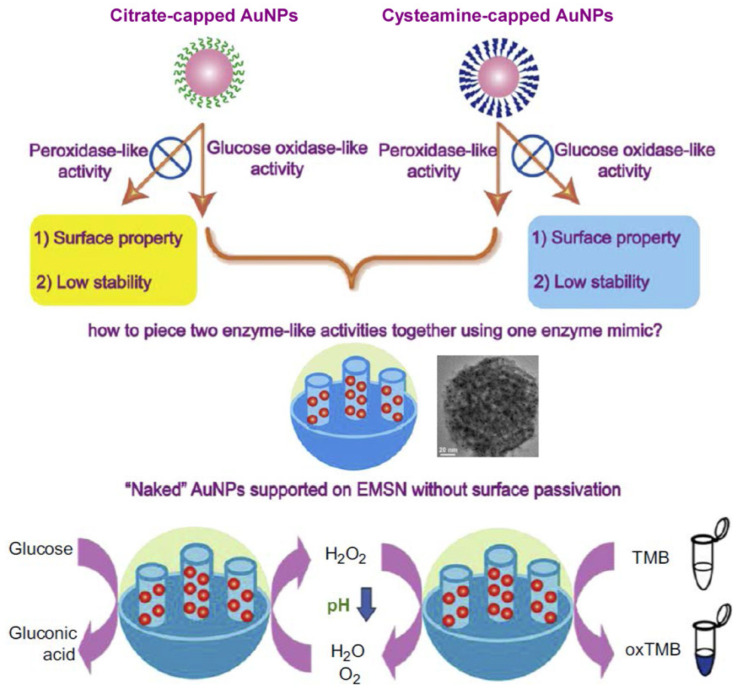
Schematic illustration of the design of EMSN-AuNPs showing both GOx- and peroxidase-like activities to trigger cascade catalysis (Reprinted with permission from Ref. [106]. Copyright 2013, Elsevier).

As a noble metal nanozyme, nano-sized Au can exhibit various artificial enzyme-like talents [104]. Typically, “naked” or “non-naked” AuNPs can provide both GOx- and peroxidase-like catalytic activities under similar or the same condition [36,37,105,106]. Previously, cysteamine-capped AuNPs were reported to provide peroxidase-like activity but not GOx-like, whereas citrate-capped AuNPs showed GOx-like catalytic activity but not peroxidase-like. The single-component AuNPs with dual enzyme-like catalytic functions were not realized. To solve such a dilemma, Qu’s group designed “naked” AuNPs that were supported on an expanded mesoporous silica support (EMSN) (Figure 6), thus obtaining significant dual enzyme-like activities [106]. In detail, the “naked” AuNPs exposed rich surfaces and sites for catalytic reactions, and the porous support not only stabilized these “naked” AuNPs well, but also offered abundant pores and channels for mass diffusion. When glucose was fed, the designed EMSN-AuNPs first catalyzed the redox reaction between glucose and dissolved O_2_ at neutral pH to produce gluconic acid and H_2_O_2_, and then the formed gluconic acid could decrease the pH value, leading to the activation of the AuNP-catalyzed redox reaction between H_2_O_2_ and TMB under acidic conditions. Such a self-activation cascade catalytic system driven by the dual enzyme-like EMSN-AuNPs shows great promise in biomedicine and sensing.

**Figure 7:**
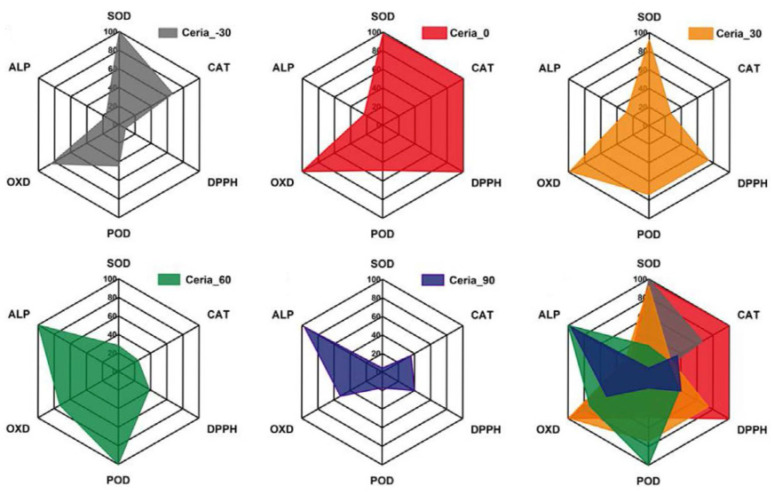
Ceria synthesized at various temperatures shows different multi-enzyme-mimetic activities (Reprinted with permission from Ref. [108]. Copyright 2021, Royal Society of Chemistry).

With the presence of the Ce^4+^/Ce^3+^ redox pair and rich vacancies, ceria (CeO_2_) is able to offer oxidoreductase-like activities as well as a hydrolase-like feature [107,108]. Interestingly, Wei’s group found that ceria could present versatile catalytic activities, including superoxide dismutase (SOD), catalase (CAT), oxidase (OXD), peroxidase (POD) and alkaline phosphatase (ALP), and the synthetic temperature of ceria made a significant impact on its multi-enzyme-mimetic activities [108]. They used a common wet-chemical method performed at various temperatures to gain the enzyme mimic, and tested the POD and OXD activities in a medium acidic environment, whereas the ALP and CAT activities were tested under alkaline conditions. As found in Figure 7, the obtained Ceria_0 showed the highest activities in SOD-mimicking, CAT-mimicking, DPPH-scavenging and OXD-mimicking. In contrast, the worst activities in SOD-mimicking, CAT-mimicking, DPPH-scavenging, POD-mimicking and OXD-mimicking were found in the obtained Ceria_90. Such a difference might be closely related to the diversities in crystal size, oxygen species and self-restoring ability exhibited in these materials.

Theoretical and experimental results reveal that, similar to Ce-based materials, Fe-containing compounds also have the potential to exhibit different enzyme-like activities [109,110]. In theory, Guo et al. combined density functional theory calculations with micro-kinetic modeling to demonstrate that Fe_3_O_4_ could exhibit the intrinsic activities of catalase, superoxide dismutase and peroxidase [110]. Three possible reaction mechanisms of the catalase-mimicking activity, namely base-like dissociative mechanism, acid-like dissociative mechanism and bi-hydrogen peroxide associative mechanism, and two possible reaction mechanisms of the superoxide dismutase-mimetic behavior, namely Langmuir-Hinshelwood mechanism and Eley-Rideal mechanism, were explored. It was identified that the acid-like dissociative mechanism and the Langmuir-Hinshelwood mechanism were, energetically, the most favorable pathways for catalase-mimicking and superoxide dismutase-mimicking, respectively.

## 4. Applications of Nanozymes with Multiple Activities in Analytical Sensing

With the rational design and rapid development of nanomaterials featuring multi-enzyme-like activities, most have been applied to biomedicine [31,32,33,34,90,94]. Besides, some of them have been used to develop advanced analytical methods and sensors [35,36,37,38,96,97,99,100,102,105,111,112,113,114,115], as summarized in Table 1. According to these examples, it is not hard to conclude that the use of multi-activity nanozymes can bring new opportunities to the analytical sensing field.

First, expanding the scope of analytes and their detection methods’ currently most of the nanozyme-involved analytical methods and sensors work based on a single catalytic reaction, which provides amplified signals for the detection of the analyte of interest. It is easy to understand that, when a nanozyme with multiple activities is used as the sensing element, different analytes can be detected using the same nanozyme, based on its different activities and responses to these analytes. As for the same analyte, one can use its various impacts on the different activities of the nanozyme to design methods for interactive detection. Multi-signal and multi-mode sensing methods for an analyte can also be realized utilizing multi-activity nanozymes, which are supposed to offer more sensitive and accurate detection. As a result, the scope of analytes as well as their detection methods can be greatly expanded with the help of multi-activity nanozymes.

**Figure 8:**
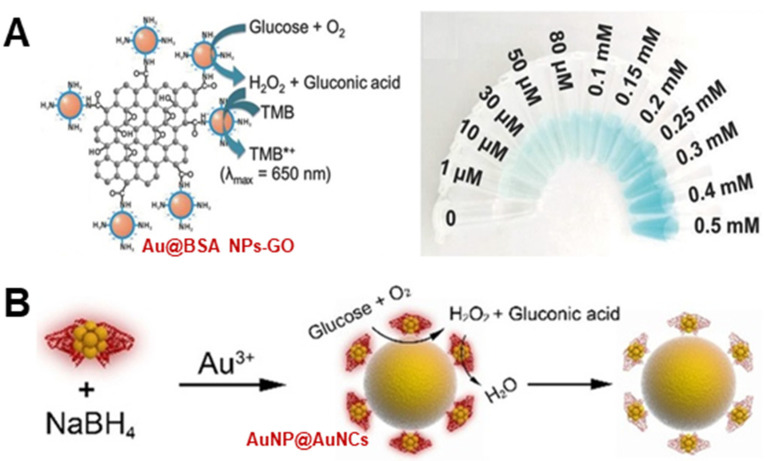
(**A**) presents the colorimetric detection of glucose using Au@BSA NPs-GO with both GOx- and peroxidase-like activities to trigger the TMB chromogenic reaction (Reprinted with permission from Ref. [36]. Copyright 2018, John Wiley & Sons). (**B**) shows AuNP@AuNCs as a self-indicative nanozyme with GOx- and peroxidase-mimetic activities for bimodal glucose detection (Reprinted with permission from Ref. [37]. Copyright 2019, John Wiley & Sons).

Second, fabricating cascade sensing systems with enhanced performance and easier operation, cascade reactions driven by a series of natural enzymes in biological systems can effectively improve the overall catalytic performance thanks to reduced intermediate decomposition, efficient mass diffusion and high local concentration. Nanozymes, as promising alternatives to these natural enzymes, not only show enzyme-mimetic activities but also provide high stability and tunable catalytic performance. In this regard, several cascade sensing systems enabled by multi-activity nanozymes have been successfully fabricated [35,36,37,38,105]. For instance, Zhang et al. established a one-pot bio-enzyme-free strategy for the colorimetric determination of glucose utilizing the dual enzyme-like activities of Au@BSA-GO [36], where the oxidation of glucose to produce H_2_O_2_ and the detection of H_2_O_2_ via the catalyzed TMB chromogenic reaction were simultaneously performed on the bi-active catalyst (Figure 8A). When increasing concentrations of the analyte glucose were fed, the cascade reaction solution provided a gradient color change, which could even be identified by the naked eye. With the cascade sensing system driven by the Au@BSA-GO with both GOx- and POD-mimetic activities, a wide range of the analyte was quantitatively detected, with a limit of detection down to 0.6 μM. More interestingly, Chen et al. designed a self-indicative Au-based nanozyme for the dual-mode determination of H_2_O_2_ and glucose [37]. In their design, a composite of Au nanoparticles encapsulated by Au nanoclusters (AuNP@AuNCs) was used as an enzyme mimic, which not only exhibited both POD- and GOx-like activities, but also presented intrinsic optical properties. As illustrated in Figure 8B, the AuNP@AuNCs first exhibited GOx-mimetic behavior to catalyze the oxidation of glucose into gluconic acid and H_2_O_2_, and the produced intermediate H_2_O_2_ could further be decomposed by the POD-like activity of the mimic to trigger the TMB color reaction, thus providing a colorimetric mode for glucose sensing. Besides, the intrinsic fluorescence of the AuNP@AuNCs was very sensitive to the level of H_2_O_2_, where H_2_O_2_ with a high local concentration could oxidize the Au^0^ species in the AuNP@AuNCs into Au^+^, thus quenching the fluorescence of the latter. Based on this principle, the analyte glucose could also be sensed via the fluorescence mode, with no addition of external signal indicators.

Despite these promising prospects, some challenges for multi-activity nanozymes applied in the analytical sensing field should not be overlooked. Since two or more enzyme-like activities can be presented under the same or similar conditions, interference from internal interplays and external factors exists. With respect to internal interference, potential competing reactions occurring on a multi-activity catalyst play the most important role. During multi-activity catalytic reactions, the same substrate may be involved and consumed, or the same intermediate may be produced, which will inevitably make an impact on qualitative and quantitative tests. Typically, both POD- and OXD-like activities can oxidize chromogenic substrates to give color signals for detection, where the unavoidable OXD-like catalytic reaction will affect the detection of an analyte based on the POD-mimetic activity. Another example for internal interference caused by multi-activity nanozymes is related to H_2_O_2_-mediated analytical methods and sensors. It is known that the generation of H_2_O_2_ and its further conversion catalyzed by peroxidase provide the foundation for the sensing of various analytes including glucose, cholesterol and alcohol, while the CAT-like feature exhibited in the multi-activity nanozyme will inevitably consume the intermediate H_2_O_2_ by converting it into H_2_O and O_2_, although the reaction usually occurs more successfully in a neutral or alkaline environment. Unavoidable consumption of the species triggered by the competing reaction not only reduces the detection sensitivity but also affects the reliability. All the above cases remind us of the importance of avoiding potential competing reactions when using multi-activity nanozymes to design analytical methods. As for external interference, complex factors exist, possibly having impact on the multi-activity nanozyme used. In comparison with single-activity materials, multi-activity nanozymes are more prone to be affected by these external factors. Especially in cascade catalytic and sensing systems, the external factor affecting one activity of nanozymes will have a great influence on all the reactions. As a result, more factors potentially affecting the sensing systems should be taken into consideration.

## 5. Conclusions

It is unquestionable that the nanozyme field is undergoing a rapid development period because of increasing interest and attention, drawn by its attractive merits. Especially, nanozymes featuring multi-enzyme-like catalytic activities are finding promising applications in biomedicine and sensing. The use of multi-activity nanozymes not only greatly expands the scope of analytes as well as their diversified sensing methods, but also enables the fabrication of cascade catalytic and sensing systems with enhanced performance and convenient operation, implying their great promise in the analytical sensing field. Despite these prospects, more effort is required to further advance the development of multi-activity nanozymes and their applications in the analytical sensing field, including expanding the types of multi-activity materials, as well as the types of multiple activities, rationally regulating the interactions between multiple activities, and developing new analytical methods and sensors based on the multiple activities of nanozymes:

(1) Types of multi-activity materials. Although the materials that can be used as enzyme mimics have been expanded to various transition metals, noble metals and carbon-based materials from initial Fe_3_O_4_ particles, the types of multi-activity nanozyme materials developed are still very limited currently. To expand their potential applications and better employ them in biochemical sensing, more materials with multiple enzyme-like activities are expected to be designed and developed in future;
(2) Types of multiple activities. Currently, most applications of multi-activity nanozymes are performed on the basis of their oxidoreductase-like activities, including POD, OXD, CAT and SOD. In fact, there are several other types of enzyme activities that artificial materials can simulate according to bionics. Integrating other types of enzyme-like activities with these oxidoreductase-like activities will break new ground for various applications, not limited to analytical sensing.
(3) Rational regulation of the interactions between multiple activities. Rational design of hybrid nanozymes with multi-enzyme-mimetic activities and accurate regulation of the interactions between these multiple activities, including proximity effect, confinement effect and mass diffusion path, are significant in thedevelopment of high-efficiency cascade catalytic and sensing systems. Strictly, only materials featuring multi-enzyme-like activities can be served as “all-in-one” catalysts, thus providing high-performance cascade reaction systems for biomedical and sensing applications.
(4) Development of new analytical methods and sensors using the multiple activities of nanozymes. As can be seen from Table 1, although a number of materials have been reported to have multi-enzyme-like activities, their applications in the analytical sensing field is still very limited. More seriously, a majority of these reported activities are not rationally utilized to serve analytical applications. In this regard, further effort is necessary to design and develop more detection methods and sensors to advance the nanozyme-involved sensing field.
(5) Integration of multiple activities with multiple functions in a nanozyme. Because of the nature of the nanoscale material, nanozymes not only present the enzyme-like catalytic feature but can also provide other optical, electrical and magnetic characteristics. For instance, metal nanoclusters not only show a fluorescent property, but have also been demonstrated to present enzyme-like behaviors [116,117,118]. Combining the catalytic activity of these nanoclusters with their optical features will open up a new window for nanozyme-based analytical sensing.

To sum up, increasingly scientists are dedicating to designing nanozymes with multiple catalytic activities for sensing applications, because these multi-activity nanozymes can bring benefits to this field. Several potential challenges from both internal and external interference should not be overlooked when using a multi-activity nanozyme to fabricate analytical methods and sensors. This requires the rational design of nanozymes with the desired activities but without interfering features. More attention should also be paid to how to take full advantage of these desired activities to fabricate sensing methods. With the trend of multi-activity nanozymes used in biochemical detection, it is believed that increasing nanozymes with more than one catalytic feature will be designed and developed in the near future, and more advanced detection strategies and sensors with good performance as well as simple operation will be fabricated based on these nanozymes.

## Figures and Tables

**Table 1:** Multi-activity nanozymes applied in the analytical sensing field.

Nanozyme	Potential Activity	Activity Used	Analyte	Detection Mode	Real Sample	Ref.
AuPd-NE	GOx, POD	GOx and POD	glucose	colorimetric	human serum	[35]
CuS	POD, CAT, AAO, SOD	AAO	ACP	fluorescence	human serum	[102]
Co_1.5_Mn_1.5_O_4_	LAC, POD, OXD, CAT	LAC; OXD	catechol; hydroquinone	colorimetric	water samples	[111]
PVP/IrPt	POD, OXD, CAT	POD	glucose	colorimetric and fluorescence	none	[99]
CoFe_2_O_4_/H_2_PPOP	OXD, POD, CAT, SOD	OXD	Cr(Ⅵ)	colorimetric	water samples	[112]
Co_3_O_4_	OXD, POD, CAT, SOD	OXD; POD	ACP; H_2_O_2_	colorimetric	human serum	[113]
Pt/WO_2.72_	POD, CAT	POD	H_2_O_2_ and glucose	colorimetric	human serum	[97]
FeO_x_@ZnMnFeO_y_@Fe-Mn	POD, OXD, CAT	POD; OXD	citric acid and norfloxacin; gallic acid	colorimetric	juice	[96]
AuNP@AuNCs	GOx, POD	GOx and POD	glucose	colorimetric and fluorescence	none	[37]
GNE-based AuNPs	GOx, POD	GOx and POD	glucose	colorimetric	none	[105]
ZIF-67/Cu_0.76_Co_2.24_O_4_	POD, GPx, SOD, CAT	CAT	3,4-dihydroxyphenylacetic acid	electrochemical	rat brain	[100]
Au@BSA-GO	GOx, POD	GOx and POD	glucose	colorimetric	none	[36]
Fe_3_O_4_-Au@MS	GOx, POD	GOx and POD	glucose	colorimetric	none	[38]
Fe_3_C/C	POD, OXD, CAT	OXD	glutathione	colorimetric	none	[114]
Cu_3_V_2_O_7_(OH)_2_	POD, OXD, LAC	OXD	glutathione	colorimetric	human serum	[115]

POD: peroxidase; OXD: oxidase; CAT: catalase; LAC: laccase; GPx: glutathione peroxidase; AAO: ascorbic acid oxidase; GOx: glucose oxidase; SOD: superoxide dismutase.

## Abbreviations

2-mIM	2-methylimidazolate
AAO	ascorbic acid oxidase
ABTS	2,2-azino-bis(3-ethylbenzothiazoline-6-sulfonic acid)
ACP	acid phosphatase
ALP	alkaline phosphatase
AuNP@AuNCs	Au nanoparticles encapsulated by Au nanoclusters
AuNPs	gold nanoparticles
BA	benzoic anhydride
BrPE	2-bromo-1-phenylethanone
BSA	bovine serum albumin
CAT	catalase
DAB	di-azo-amino-benzene
DMNP	dimethyl 4-nitrophenyl phosphate
EMSN	expanded mesoporous silica support
Fe_3_O_4_-Au@MS	mesoporous silica-coated Fe_3_O_4_-Au
GMP	guanosine monophosphate
GO-COOH	carboxyl-modified graphene oxide
GODs	graphene quantum dots
GOx	glucose oxidase
GPx	glutathione peroxidase
hCAII	human carbonic anhydrase II
HRP	horseradish peroxidase
LAC	laccase
MNP	magnetic nanoparticles
MOF	metal-organic framework
OPD	*o*-phenyl-enediamine
OXD	oxidase
PH	phenyl-hydrazine
*p*NPA	*p*-nitro-phenyl acetate
POD	peroxidase
SOD	superoxide dismutase
TA	tereph-thalic acid
TCPP	tetra-carboxylic phenyl porphyrin
TMB	3,3′,5,5′-tetramethylbenzidine
V-HPO	vanadium-dependent halo-peroxidase
ZIFs	zeolitic imidazolate frameworks
